# The role of the differential outcomes procedure and schizotypy in the recognition of dynamic facial expressions of emotions

**DOI:** 10.1038/s41598-024-52893-9

**Published:** 2024-01-28

**Authors:** Antonio González-Rodríguez, Ángel García-Pérez, Marta Godoy-Giménez, Pablo Sayans-Jiménez, Fernando Cañadas, Angeles F. Estévez

**Affiliations:** 1 Department of Psychology, University of Almería, Ctra Sacramento S/N, La Cañada de San Urbano, CP: 04120 Almería, Spain; 2 CEINSA Health Research Centre, University of Almería, Almería, Spain; 3 CIBIS Research Centre, University of Almería, Almería, Spain

**Keywords:** Human behaviour, Emotion, Social behaviour, Operant learning

## Abstract

Emotional facial expression recognition is a key ability for adequate social functioning. The current study aims to test if the differential outcomes procedure (DOP) may improve the recognition of dynamic facial expressions of emotions and to further explore whether schizotypal personality traits may have any effect on performance. 183 undergraduate students completed a task where a face morphed from a neutral expression to one of the six basic emotions at full intensity over 10 s. Participants had to press spacebar as soon as they identified the emotion and choose which had appeared. In the first block, participants received no outcomes. In the second block, a group received specific outcomes associated to each emotion (DOP group), while another group received non-differential outcomes after correctly responding (NOP group). Employing generalized linear models (GLMs) and Bayesian inference we estimated different parameters to answer our research goals. Schizotypal personality traits did not seem to affect dynamic emotional facial expression recognition. Participants of the DOP group were less likely to respond incorrectly to faces showing Fear and Surprise at fewer intensity levels. This may suggest that the DOP could lead to better identification of the main features that differentiate each facial expression of emotion.

## Introduction

Emotions play an important role in daily-life social interactions. Being able to correctly decode facial expressions is essential for building successful relationships and for adaptative social functioning^1–3^ since these expressions are one of the most common ways of communicating the emotional state of an individual. It is noteworthy that this ability has also been associated with important features, such as parenting^4,5^, quality of life^6^, work functioning, and independent living in people with mental illness^7^. Therefore, fostering an accurate emotional facial expression recognition would thus be desirable, and methods that may ameliorate the possible deficits in this ability are worth investigating. The differential outcomes procedure (DOP) consists in an easy-to-apply and free-cost procedure that has been shown to improve learning rates and accuracy^8^. This procedure involves the association of a specific outcome with each stimulus–response sequence or stimulus to-be-recognised. In opposition to the non-differential outcomes procedure (NOP; the reinforcer is presented randomly or non-specifically after correct responses), when we use the DOP we always present the same reinforcer when a correct response to a specific stimulus is made. Some studies have suggested that this procedure could be useful to improve performance in specific tasks that may be affected in some populations, like those involving visuospatial working memory^9^, visual memory^10^, and the recognition of facial identities^11^ in people diagnosed with Alzheimer’s disease or alcohol dementia^12^. However, as far as we know, there is only one study that explored the effect of the DOP on the recognition of static emotional facial expressions^13^, suggesting that this procedure could also be useful for this purpose and might lead a better and faster recognition of emotion in facial expressions. Nevertheless, this study showed some limitations concerning sample sizes, featured static stimuli, and asserted that it was a preliminary study and their results should be interpreted as such.

The literature consistently shows that some clinical populations may be impaired in their ability to recognise emotional facial expressions^14,15^. As an example, people diagnosed with schizophrenia have been suggested to present a worse general emotional facial expression recognition, with effect sizes ranging from moderate to severe^16,17^, when they are compared with a non-clinical sample. Furthermore, other variables, such as the levels of some personality traits, may also affect this ability for which literature is, at the moment, inconclusive. For this research, we will be mainly focused on schizotypy, which could be described as a set of traits that resemble those symptoms present in people diagnosed with schizophrenia, although of minor intensity, and that may be present in both clinical and non-clinical populations^18,19^ at different degrees of severity^20–22^. Literature also indicates that people presenting high levels of these traits could be at risk for developing psychosis spectrum disorders^23,24^.

High levels of schizotypal personality traits have been apparently associated with emotional processing deficits^25,26^. Concretely, some studies exploring emotional facial expression recognition in people with varying levels of these traits have found that the higher these levels, the lower the accuracy in a computerized task^27,28^, but other studies have not found this effect^29–32^. The multidimensional operationalization of schizotypy provides an opportunity that may be useful for identifying the key features of schizophrenia^33,34^, so expanding knowledge in this field could be directly beneficial for clinicians and researchers. Besides, for those interested in individual differences, schizotypal personality traits may not be only regarded as a clinical condition, but also a personality dimension aside from schizophrenia^35^, which study could be beneficial for social and affective sciences^26^. As impairments in the recognition of facial expressions of emotion can be expected in people with higher levels of schizotypal personality traits^36^ and the DOP is increasingly beneficial as discrimination difficulty increases^8^, we can expect, if schizotypal personality traits have an effect on performance, that the DOP will provide a larger enhancement for those participants with higher schizotypal personality levels.

Importantly, several variables may be considered when an emotional facial expression recognition study is designed. Most studies carried out to assess this ability employ computerized tasks in which participants must recognise an emotion expressed in static pictures of faces, although in real-life situations emotional facial expressions are not seen as static pictures, but rather as dynamic expressions that change over time^37^. Another important variable to consider is the intensity at which the emotional expressions will be presented, which may improve the probability of correctly identifying the emotion as intensity increases. People diagnosed with schizophrenia do not seem to be benefited from this effect, however^38^. Lastly, some studies have employed virtually generated faces instead of real pictures of faces. This methodology allows for the manipulation of facial features to reliably represent emotions and has shown similar effectiveness compared with real pictures in healthy^39^ and clinical populations^40^. In our study, we aim to maintain high controllability of the presented stimuli as well as to adopt a better representation of real-life situations, so we will employ virtually generated faces which will dynamically modify the intensity of the presented emotion in the sample stimuli.

Considering all these issues, the current study presents two main goals. On the one hand, we want to explore, employing virtually generated faces, if the DOP may be beneficial for improving the ability to recognise dynamic emotional facial expressions and, on the other hand, to further explore whether schizotypal personality traits may lead to any impairment regarding this ability. For testing these aspects, we will focus on how the intensity of the facial expression of emotion shown in a face may modify the probability of making a correct response during the task and on the amount of learning produced during the Based on previous literature employing the DOP, we expect this effect to be more notable for those facial expressions of emotion or conditions that present a greater difficulty^41,42^.

## Methods

### Participants

A total of 183 undergraduate students (141 women) with normal or corrected-to-normal vision that were recruited from the University of Almeria by convenience or snowball sampling participated in the study. Only participants who reported no history of psychiatric illness and whose ages ranged from 18 to 32 years (*M* = 20.5, *SD* = 2.7) were included. Participants that were enrolled in the Bachelor’s Degree in Psychology received one course credit for their voluntary collaboration, and all participants participated in a raffle with the prizes that were displayed in the images shown as reinforcers during the task (see “Instruments”). Written informed consent was required from all participants, and they were informed that they could cease their participation at any moment. This study was approved by the local Bioethics Committee in Human Research and was conducted following the Declaration of Helsinki.

### Instruments

#### Dynamic emotional facial expression recognition task

In our experiment, we generated short video clips (10 s) in which a human face was shown gradually morphing its emotional expression from neutral to one of the basic emotions (Anger, Disgust, Fear, Happiness, Sadness, or Surprise^43^) at full intensity. Several steps were followed to achieve this. First, we chose pictures of five Caucasian men and five Caucasian women from the racially diverse affective expression (RADIATE) face stimulus set^44^ that showed a neutral expression. Then, we employed the software Facegen Modeller^45^ to import these neutral faces, creating a virtual version of them. This software allows changing the facial configuration of the target face by modifying the desired Action Units (AUs), which consist of movements of muscles that change the expression of the face and may reliably represent prototypical basic emotions when combined.

Following the Facial Action Coding System (FACS) Investigator’s Guide^46^ we modified the AUs of these faces to show the six basic emotions. As one emotion may be expressed by several combinations of AUs, data from a previous unpublished study was used to determine which facial configuration was going to be chosen. In this study, 83 undergraduate students (50 women) rated from 0 (not representative at all) to 10 (totally representative) how each of the 51 combinations of AUs represented each basic emotion. Those facial configurations with the highest mean ratings for a specific emotion (see Supplementary Material) were employed as the prototypical facial configuration for that specific emotion. The chosen AUs were: 4, 5, 7, 10, 22, 23, and 26 for Anger; 10 for Disgust; 1, 2, 4, 5, 20, and 26 for Fear; 6 and 12 for Happiness; 1, 4, 15, and 17 for Sadness; and 1, 2, 5, and 27 for Surprise. Then, we used Abrosoft FantaMorph 5^47^ to morph these faces from a neutral expression to each of the abovementioned combinations of AUs, generating six videos, one per basic emotion, for each of the 10 models. The videos lasted 10 s and contained 100 frames.

Using the free software PsychoPy^48^ we designed our task, which consisted of two blocks. In the first block, participants watched the video clips that showed the faces morphing from neutral to one of the basic emotions in a random order, and they were instructed to press the spacebar as soon as they recognised the facial expression of emotion that was shown. Once participants pressed the spacebar, or after the 10 s that lasted the video, the video disappeared and participants saw labels corresponding to each basic emotion. Participants had to choose the label of the emotion they believed was just shown (e.g., Anger). This way, we could obtain both the reaction times when the response was made, which would be equivalent to the intensity shown in the emotion at that moment (from 0% at 0 s to 100% at 10 s), and the response itself to evaluate the accuracy of our participants. Faces showing each emotion were shown 10 times, one per each of the chosen models of the RADIATE face stimulus set^44^, so the total number of trials was 60 per block. The second block was identical to the first block, except now the participants received feedback after their correct responses. Concretely, they saw the picture of one of the possible prizes that were raffled after the experiment, which were a keychain, a pen drive, an earphone adapter, a playing dice, a mug, and a book with riddles. The duration of the experiment was approximately 15 min.

#### Schizotypal personality questionnaire

For the assessment of schizotypal personality trait levels, we used the schizotypal personality questionnaire (SPQ)^49^. This tool has shown good psychometrical properties in different populations and cultures^50^. It comprises 74 items distributed across nine subscales, each containing seven items that are answered dichotomously, with “Yes” responses yielding a score of 1 and “No” responses yielding a score of 0. Literature suggests that these traits are well explained by a three-factor model^51–53^. The Cognitive-Perceptual factor (α = 0.85, this study) has a score ranging from 0 to 33 and integrates the four subscales Odd Beliefs or Magical Thinking, Unusual Perceptual Experiences, Ideas of Reference, and Suspiciousness. The Interpersonal factor (α = 0.87, this study) has a score ranging from 0 to 33 and integrates the four subscales Social Anxiety, No Close Friends, Constricted Affect, and Suspiciousness. Lastly, the Disorganized factor (α = 0.82, this study) has a score ranging from 0 to 16 and integrates the two subscales Odd or Eccentric Behaviour and Odd Speech. The reliability for each factor was calculated on the set of items that compose each factor score. For this study, we used the Spanish-validated version of the SPQ^54^.

### Procedure

When the sample was collected there were still several limitations involving physical contact and space usability due to the Severe Acute Respiratory Syndrome Coronavirus 2 (SARS-CoV2), so we decided to assess our participants online. To recruit participants, we announced on the online learning platform for the bachelor’s degree in psychology that an experiment involving the recognition of emotions was going to be performed. The announcement stated that they would receive one course credit for participating and that they could have other two acquaintances that were also undergraduate students do the experiment in order to receive one additional course credit. All participants read the instructions and the data treatment ethics, and provided consent to participate in the study, agreeing to the use of their data only for scientific purposes before starting the experiment. They were also informed that they would participate in a raffle which could award them one of the abovementioned prizes. Finally, they had to contact the research team via e-mail to participate. After they had contacted the email address, they were sent the informed consent and were asked to return it correctly signed.

Once a fulfilled informed consent was received by a participant, an email was sent to them with some guidelines they should follow to correctly do the experiment. These guidelines stated that they should (1) do the experiment on a desktop computer using a mouse and keyboard, (2) be in a room with adequate lighting conditions, (3) avoid stopping or taking a rest in the middle of the experiment, (4) do the experiment when they had no distractions and with their phone turned off, (5) deactivate their antivirus to avoid incompatibilities with the task, (6) and to not do the experiment if they were tired or in any altered emotional state. They were also provided with a link that led them to one of the two online versions of the experiment, hosted in Pavlovia (https://www.pavlovia.org). Both versions differed in the way outcomes were provided during the second block of the task (explained below), which depended on the group the participant had been randomly assigned, that could be the DOP group (*n* = 88; 64 women) or the NOP group (*n* = 95; 77 women). Mean years age of participants was similar in both groups (DOP mean age = 20.8, SD = 2.8; NOP mean age = 20.2, SD = 2.7).

**Figure 1:**
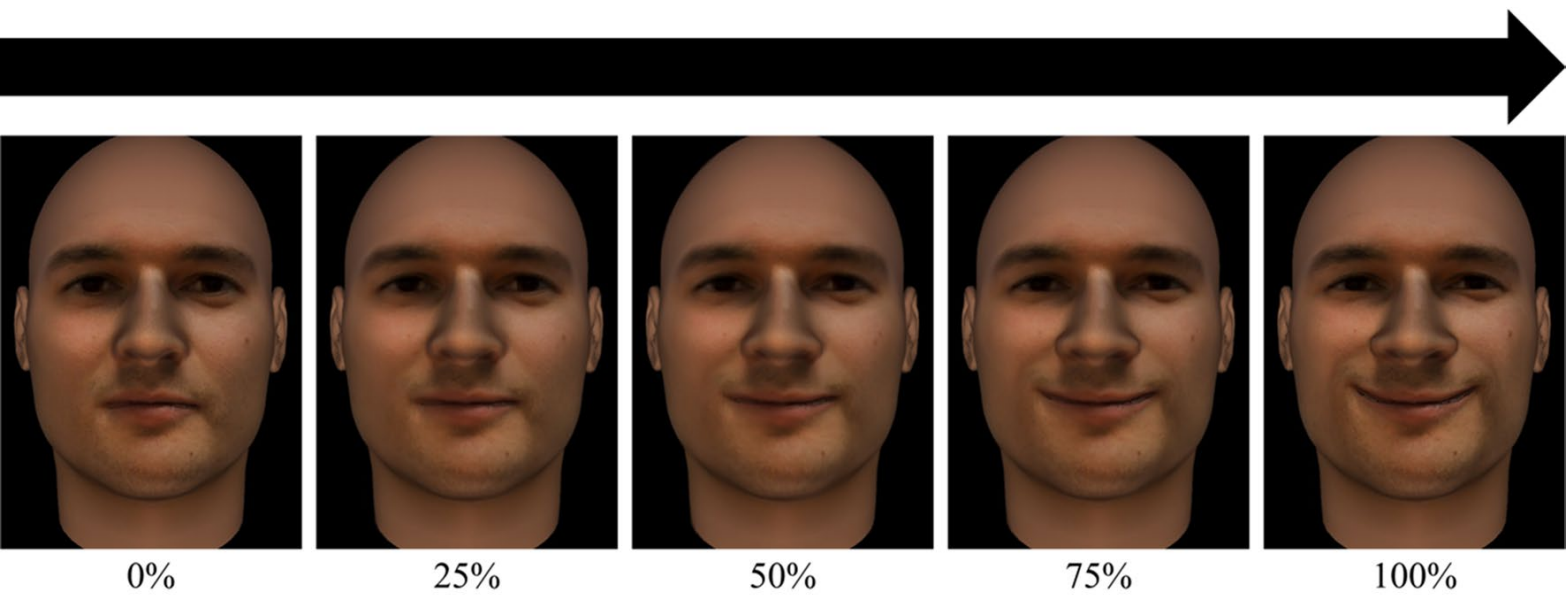
Sample stimulus showing the emotion of happiness at different frames. *Note.* Only 5 frames are shown in this figure to represent intensity of the emotion at different thresholds. In the real task, there could be up to 100 frames in which the intensity of the emotion increased gradually.

The experiment began by showing the instructions to the participants along with a diagram representing a trial of the task. After they pressed any key, a practice trial began. They could repeat this trial as many times as they want until they were prompted to select that they had correctly understood the task. Once they confirmed the task was understood, the first block of the task began. In each trial, they were shown one of the created videos from the faces of the 10 chosen actors morphing from neutral to each of the six basic emotions at full intensity in random order until they saw them all. An example of a sample stimulus at different intensities is represented in Fig. 1.

In the first block, a fixation cross appeared for 1000 ms after an emotion label was chosen as the response. Subsequently, they performed the second block, which was similar to the previous one with the difference that participants now saw pictures of the possible prizes after their correct responses for 1000 ms along with the sentence “You can win a (*name of the reinforcer*)!”. If the participant was assigned to the DOP group, the outcome was always the same when they correctly answered a specific emotion. This means that, for example, after correctly identifying the emotion “Happiness”, they always saw the picture of the keychain, with reinforcers randomly assigned to each stimuli emotion among participants. On the other hand, participants in the NOP group could see any reinforcer after any correct response, so the same emotion was always followed by any reinforcer. If participants made an incorrect response, they saw a blank screen for 1000 ms.

Participants were also sent a link to an online version of the SPQ, which was hosted in LimeSurvey (https://www.limesurvey.org/) and were instructed to complete it after the task had finished. If one week later they had yet not completed the questionnaire, they were asked again to complete it. In the end, several people (*n* = 74) did not complete the questionnaire.

### Data analysis

We were interested in exploring the effects of schizotypal personality trait levels and the differential outcomes procedure for each of the stimuli emotions, so we decided to employ Generalized Linear Models (GLMs). The use of GLMs allows for flexible estimations that may adapt to the particular needs of each situation. Firstly, we are interested in the effect that the intensity shown by the face may exert on the probability of making a correct response. Secondly, we wanted to explore the learning in this task by estimating whether the probability of correctly responding may change across trials generating a learning curve. Furthermore, we explored whether learning was different when we compare the different procedures (DOP and NOP). These parameters were estimated over the whole sample in every condition for every presented emotion. For participants whose schizotypal personality traits were assessed (*n* = 109; 58 from the DOP group and 51 from the NOP group), additional parameters that estimated their effect on the probability of making a correct response, as well as moderation effects with the abovementioned parameters, were included. As there are many contrasts to be made we decided to employ a fully Bayesian approach since all this information may be extracted from the same posterior distribution, which implies that data may be explored from multiple perspectives without affecting the statistical inference process^55^.

For the estimation of the differences between parameters in each condition, we generated posterior distributions from the subtractions between parameters in each sample of the posterior of the to-be-compared conditions. The Highest Density Interval (HDI), which is the most credible span of values that cover 95% of the posterior distributions of the parameters of interest or the differences between parameters will be used, along with a Region Of Practical Equivalence (ROPE), to make statistical inferences based on our data. The ROPE is a range around specific values of interest (e.g., zero when we are interested in the differences between means). Following Kruschke^56^, if the HDI completely excludes the ROPE, we will conclude that values inside the ROPE are not credible, and if the HDI overlaps with the ROPE we will withhold our decision. To ease readability, the specific ROPEs employed when making contrasts for each parameter are detailed in their respective sections within Results. The employed dataset is publicly available at the Open Science Framework (OSF) repository (https://osf.io/ygpx9/?view_only=dbdbe28eb8554451bf90e4ff69f357c4).

#### Model for participants who did not complete the SPQ

For those who did not complete the SPQ, the model was defined as follows:

1 $$P_{\left[ i \right]} { }\sim {\text{ Binomial}}\left( {{ }1{ },{ }p_{\left[ i \right]} } \right)$$

2 $${\text{logit}}(p_{\left[ i \right]} ) = { }\alpha_{{\left[ {cond24\left[ i \right]} \right]}} + { }\beta_{{RT\left[ {cond18\left[ i \right]} \right]}} {*}\left( {\log \frac{{{\text{RT}}_{\left[ i \right]} }}{10}} \right) + { }\beta_{{Trial\left[ {cond18\left[ i \right]} \right]}} *\left( {\frac{{10 - {\text{Trial}}_{\left[ i \right]} }}{9}} \right)^{e}$$

3 $$\alpha_{{\left[ {cond24\left[ i \right]} \right]}} { }\sim { }N\left( {0,{ }1.5} \right)$$

4 $$0 < \beta_{{RT\left[ {cond18\left[ i \right]} \right]}} { }\sim {\text{ HalfNormal}}\left( {0,{ }1} \right)$$

5 $$\beta_{{Trial\left[ {cond18\left[ i \right]} \right]}} { }\sim { }N\left( {0,{ }1} \right)$$

The symbol “~” means “distributed as”. Binomial(x, y) indicates a binomial distribution with *n* = x and *p* = y; *N*(x, y) indicates a normal distribution with *μ* = x and *σ* = y; and Half-Normal(x, y) indicates a distribution including only the positive values of a normal distribution with *μ* = x and *σ* = y. Equation (1) is the likelihood of the model, with *P* taking either a “1” or a “0” value if the case [*i*], which identifies each trial of each participant, is a correct or an incorrect response, respectively.

Equation (2) is the generalized linear model that estimates the probability of making a correct response in each case [*i*]. A *logit link* function is used to map the linear space of the model (-∞, ∞) with the non-linear space of *p* (0, 1), which is a common approach when working with binomial GLMs^57^. The first parameter *α* represents the intercept of the equation as the log-odds in each specific condition indicated by the subscript cond24[*i*]. In our experiment, we have two groups (DOP and NOP), each with two feedback conditions (No Feedback and Feedback) and with stimuli showing six basic facial expressions of emotions (Anger, Disgust, Fear, Happiness, Sadness, and Surprise) to recognise (called emotion hereafter), therefore, this parameter may present 24 different values depending on the case [*i*].

The second parameter *β*_*RT*_ is a penalty parameter that represents the effect that the reaction time (equivalent to the intensity of the facial expression of emotion when the response is made) may exert on the probability of making a correct response in each case [*i*], with larger values indicating a larger penalty for not seeing the emotion at full intensity in each condition indicated by the subscript cond18[*i*]. In this case, it is not expected that both groups will behave differently concerning penalties when feedback is not provided, so we have three feedback conditions (NoFeedback, DOP feedback, and NOP feedback) for each of the abovementioned six emotions, so there are 18 possible values for this parameter depending on the case [*i*]. This parameter multiplies the natural logarithm of the Reaction Time (RT) in seconds in each case [*i*], divided by 10, so 0 would indicate a response after 0 s (0% intensity of the shown emotion) and 1 would indicate a response after 10 s (100% intensity of the shown emotion). This transformation was made because the natural logarithm of 0 is − ∞ (correctly identifying an emotion should be impossible when it is presented at 0% intensity for reasons other than randomness), and the natural logarithm of 1 is 0 (there would be no penalty when the emotion is shown at 100% intensity), and we expect that increments in penalty should be lower as reaction time increases (there should be a larger difference in penalty for responding after 1 s instead of after 2 s, than for responding after 9 s instead of after 10 s).

The third and last parameter *β*_*Trial*_ accounts for the learning curve of each condition. For the same reasons as the previous parameter, 18 possible values are allowed in this parameter depending on the case [*i*]. As the formula is expressed, the value of this parameter would also indicate how the performance in the first trial of a condition differs from the last trial of that specific condition, expressed as the difference in log-odds. The reason is that when Trial_[i]_ = 1, the expression in parentheses multiplied by this parameter evaluates to 1 (10–1 = 9; 9/9 = 1; 1^e^ = 1) and when Trial_[i]_ = 10 it evaluates to 0 (10–10 = 0; 0/9 = 0; 0^e^ = 0). This expression in the parentheses is exponentiated to *e* because we assumed that learning would occur faster in the first trials and will reach an asymptote in the later trials.

As explained above, the parameters *β*_*RT*_ and *β*_*Trial*_ have a value of 0 when the stimuli are showing emotions expressed at full intensity and it is the last trial of each specific condition, so our first parameter *α* may serve as an estimation of the probability (expressed in log-odds) of making a correct response in the last trial of each condition when the emotion is presented at full intensity, while the *β*_*RT*_ and *β*_*Trial*_ may indicate the magnitude of the effects of the intensity of the emotion presented at the moment of the response (RTs) and the experience (number of trials concurred) on this probability.

Lastly, Eqs. (3)–(5) represent the priors of our parameters, with plausible values in their linear space^57^. For the second parameter *β*_*RT*_, we assumed a positive Half-Normal prior because, if this parameter could present negative values, it would mean that the more intense the emotion in the face is, the harder it is to recognise it, which should not be expected.

#### Model for participants who completed the SPQ

For those participants who completed the SPQ, information regarding their standardized raw scores in the different factors of the questionnaire was also employed for their estimations. In these cases, the model was modified as follows:

6 $${\text{logit}}(p_{\left[ i \right]} ) = {\text{logit}}(p_{\left[ i \right]} ) + \beta_{{CP\left[ {cond18\left[ i \right]} \right]}} *{\text{CP}}_{\left[ i \right]} + \beta_{{IN\left[ {cond18\left[ i \right]} \right]}} *{\text{IN}}_{\left[ i \right]} + \beta_{{DI\left[ {cond18\left[ i \right]} \right]}} *{\text{DI}}_{\left[ i \right]}$$

7 $$\beta_{{RT\left[ {cond18\left[ i \right]} \right]}} = \beta_{{RT\left[ {cond18\left[ i \right]} \right]}} *\left( {1 + \left( {\beta_{{CP\_RT\left[ {cond18\left[ i \right]} \right]}} *{\text{CP}}_{\left[ i \right]} + \beta_{{IN\_RT\left[ {cond18\left[ i \right]} \right]}} * {\text{IN}}_{\left[ i \right]} + \beta_{{DI\_RT\left[ {cond18\left[ i \right]} \right]}} * {\text{DI}}_{\left[ i \right]} } \right)} \right)$$

8 $$\beta_{{Trial\left[ {cond18\left[ i \right]} \right]}} = \beta_{{Trial\left[ {cond18\left[ i \right]} \right]}} {*}\left( {1 + \left( {\beta_{{CP\_Trial\left[ {cond18\left[ i \right]} \right]}} *{\text{CP}}_{\left[ i \right]} + \beta_{{IN\_Trial\left[ {cond18\left[ i \right]} \right]}} * {\text{IN}}_{\left[ i \right]} + \beta_{{DI\_Trial\left[ {cond18\left[ i \right]} \right]}} * {\text{DI}}_{\left[ i \right]} } \right)} \right)$$

9 $$\beta_{{CP\left[ {cond18\left[ i \right]} \right] }} ,\beta_{{IN\left[ {cond18\left[ i \right]} \right]}} ,\beta_{{DI\left[ {cond18\left[ i \right]} \right]}} { }\sim { }N\left( {0,{ }0.25} \right)$$

10 $$\beta_{{CP\_RT\left[ {cond18\left[ i \right]} \right] }} ,\beta_{{IN\_RT\left[ {cond18\left[ i \right]} \right]}} ,\beta_{{DI\_RT\left[ {cond18\left[ i \right]} \right]}} { }\sim { }N\left( {0,{ }0.1} \right)$$

11 $$\beta_{{CP\_Trial\left[ {cond18\left[ i \right]} \right] }} ,\beta_{{IN\_Trial\left[ {cond18\left[ i \right]} \right]}} ,\beta_{{DI\_Trial\left[ {cond18\left[ i \right]} \right]}} { }\sim { }N\left( {0,{ }0.1} \right)$$

Equation (6) is equivalent to Eq. (2) above, but with slope parameters (*β*_*CP*_, *β*_*IN*_, *β*_*DI*_) that take into account the score of each participant in each factor of the SPQ (CP = Cognitive-Perceptual factor, IN = Interpersonal factor, DI = Disorganized factor). These slopes may present different values for each of the 18 conditions explained above to explore if the possible effect of schizotypal personality traits levels in the probability of making a correct response depends on the condition. Equations (7) and (8) allow for a moderation effect of schizotypal personality trait levels in *β*_*Trial*_ and *β*_*RT*_ parameters in each of these conditions. In case any factor of the SPQ moderated the magnitude of these effects, the value of these parameters (*β*_*CP_RT*_, *β*_*IN_RT*_, *β*_*DI_RT*_, *β*_*CP_Trial*_, *β*_*IN_Trial*_, and *β*_*DI_Trial*_) would be credibly different from 0, indicating the proportional moderation they represent. If the values of these parameters were 0, *β*_*Trial*_ and *β*_*RT*_ would see no change in the magnitude of their effect since they would be multiplied by 1. Finally, Eqs. (9)–(11) represent the priors for these new parameters.

All these analyses were carried out using the free software “R”^58^ and the package “RStan”^59^ for full Bayesian statistical inference using Markov Chain Monte Carlo (MCMC) sampling. The total number of samples of the model was 10,000, using 4 chains, each of them saving 2500 samples after 1000 warmup samples. An adequate convergence of chains was evaluated using trace plots and the Gelman-Rubin test $\left( {\hat{R}} \right)$, which revealed values close to 1 for all parameters^60^.

## Results

### Dynamic emotional facial expression recognition task

**Figure 2:**
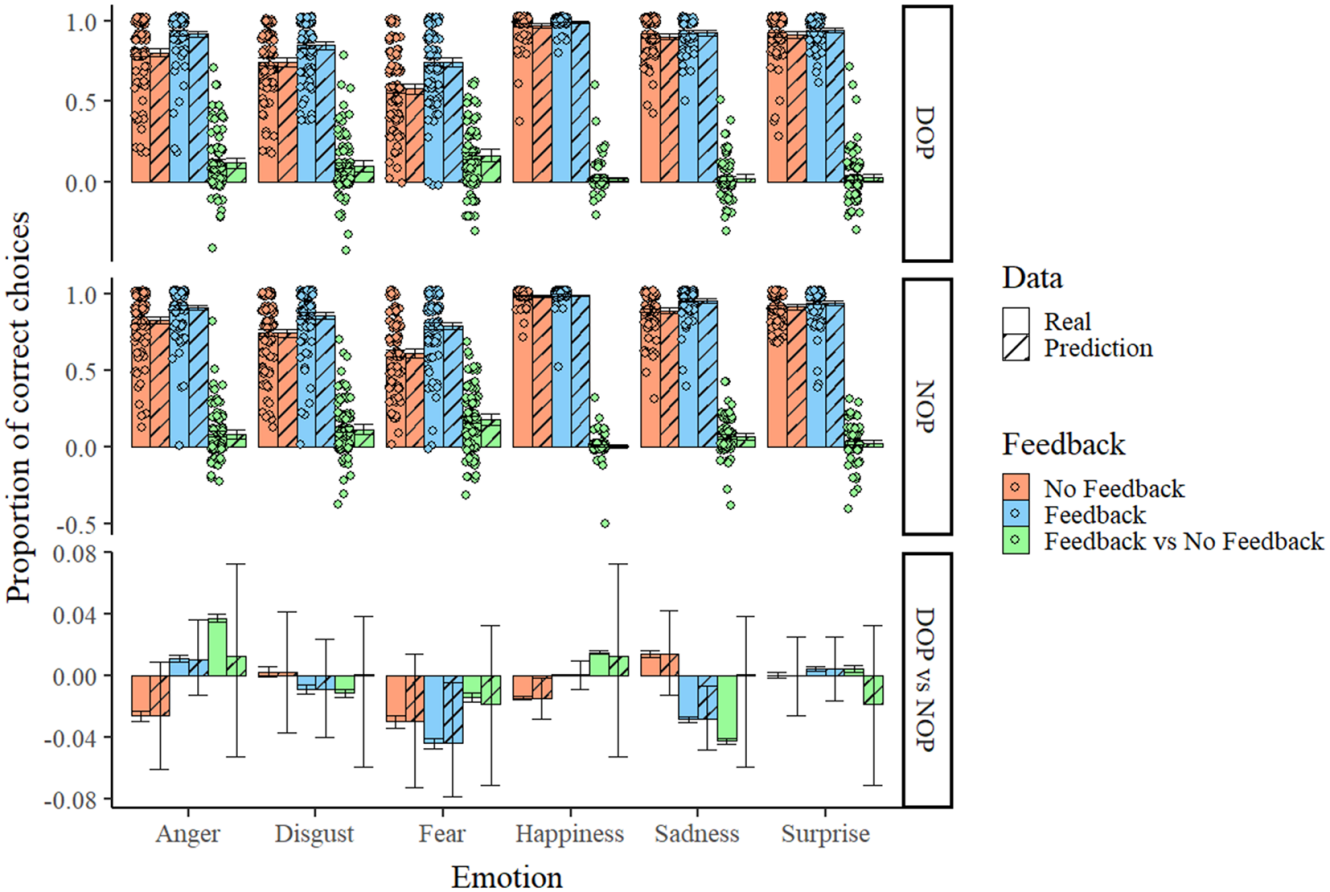
Real data (solid bars) and predictions of the model employed (stripped bars) regarding the proportion of correct choices in each condition of the task as a function of emotion, group, and feedback condition. Differences between the feedback conditions and between groups within each emotion are also depicted. For real data, individual data points are presented with some jittering to prevent overlapping. The vertical bars represent the standard error of the mean (for real data) and the lower and upper limits of the 95% HDIs (for the predictions).

Figure 2 shows the real data obtained in our task, along with the predictions of our model, suggesting an adequate fit. We will present our results focusing either on the parameters themselves or on the difference between parameters in the conditions of interest, for example, the same emotion when feedback is received in the DOP and the NOP group. Predictions and real data considering reaction time and trial number separately can be consulted in the following repository: https://osf.io/ygpx9/?view_only=dbdbe28eb8554451bf90e4ff69f357c4.

#### Probability of making a correct response when the emotion is presented at full intensity in the last trial of each condition (α parameter)

To ease interpretation, we will present our results regarding this parameter as the probability of making a correct response, as a percentage, instead of in log-odds, since this parameter may be interpreted in isolation (assuming the other parameters of the equation evaluate to 0). As our task comprised ten trials in each condition, our sensibility to detect variations in performance for each participant would be 10%. For this reason, we will establish a ROPE of half this amount (− 5.0, 5.0), which would correspond to an average change of half a correct response per individual, and we will consider as credible only those differences in which the 95% HDI completely exclude this interval. Our main interest is to explore if the DOP and the NOP may generate a different change in accuracy on any facial expression of emotion, so we will consider the change in accuracy when feedback is not received and when feedback is received for both groups in each emotion, and we will explore if the amount of this change is different between both groups. Results regarding this parameter are reported in Table 1.

**Table 1:** Means of the posteriors (and 95% HDIs) of the parameter *α* in each condition.

	DOP			NOP			DOP vs. NOP
	Feedback	No feedback	Feedback vs. no feedback	Feedback	No feedback	Feedback vs. no feedback	Feedback vs. no feedback
Anger	98.7% [97.8, 99.5]	88.4% [85.6, 91.2]	**10.3% [7.3, 13.2]**	97.7% [96.3, 98.8]	90.3% [87.8, 92.8]	7.4% [4.7, 10.4]	2.9% [1.3, 5.7]
Disgust	91.7% [88.5, 94.4]	84.5% [81.6, 87.6]	7.2% [2.8, 11.2]	91.3% [88.2, 94.2]	84.2% [81.2, 87.1]	7.1% [2.9, 11.3]	0.0% [− 5.0, 5.3]
Fear	84.5% [80.4, 88.4]	75.6% [71.9, 79.2]	8.9% [3.6, 14.5]	93.1% [90.8, 95.4]	77.6% [74.2, 80.9]	**15.5% [11.4, 19.6]**	− 6.6% [− 12.7, − 1.0]
Happiness	99.2% [98.5, 99.9]	97.7% [96.5, 98.7]	1.6% [0.2, 3.0]	99.8% [99.5, 100.0]	98.8% [98.1, 99.5]	0.9% [0.2, 1.8]	0.6% [− 0.8, 2.1]
Sadness	96.8% [94.7, 98.6]	95.3% [93.7, 97.0]	1.4% [− 1.3, 4.0]	99.0% [98.3, 99.6]	94.8% [93.1, 96.5]	4.1% [2.4, 6.0]	− 2.7% [− 5.3, − 0.1]
Surprise	95.4% [93.4, 97.3]	93.9% [91.8, 95.7]	1.5% [− 1.4, 4.2]	98.1% [97.0, 99.1]	93.8% [91.9, 95.7]	4.3% [2.0, 6.5]	− 2.8% [− 5.6, 0.3]

For each group, the third column indicates the same statistics regarding the difference between parameters when feedback is received and when it is not received. The last column compares these differences between groups.

*Note.* Credible differences in which the 95% HDI completely excludes the ROPE (− 5.0, 5.0) are boldfaced.

There were only two credible differences based on our criteria. The recognition of Anger for the DOP group when feedback was received improved (mean of the posterior = 10.3%, 95% HDI from 7.3 to 13.2%), and the recognition of Fear for the NOP group when feedback was received also improved (mean of the posterior = 15.5%, 95% HDI from 11.4 to 19.6%), both compared to the condition in which they did not receive any feedback. However, no credible differences were found between the change that both groups underwent when feedback was received and when feedback was not received in any emotion (see Table 1, last column).

#### Influence of intensity on the probability of making a correct response (β_RT_ parameter)

Regarding the *β*_*RT*_ parameter, our main aim is exploring whether the DOP and the NOP may generate different penalties in the probability of making a correct response (in log-odds) for not seeing the emotion at full intensity. As this is evaluated depending on the intensity of the expressed emotion, which may result in a severe penalty when the RT is close to 0 (the natural logarithm of 0 is − ∞), we will consider credible all the differences that exclude the value 0, so the ROPE for the differences between conditions in this parameter be just the value 0. Results reporting the values for the parameter *β*_*RT*_ are shown in Table 2.

**Table 2:** Means of the posteriors (and 95% HDIs) of the parameter *β*_*RT*_ in each feedback condition.

	Feedback condition			Differences
	No Feedback	DOP	NOP	DOP vs. NOP
Anger	0.58 [0.32, 0.85]	1.79 [1.25, 2.35]	1.24 [0.84, 1.66]	0.55 [− 0.14, 1.24]
Disgust	0.85 [0.59, 1.11]	0.74 [0.32, 1.13]	0.52 [0.19, 0.86]	0.22 [− 0.30, 0.75]
Fear	1.18 [0.88, 1.46]	0.75 [0.35, 1.13]	1.66 [1.27, 2.08]	− **0.91 [**− **1.47, **− **0.34]**
Happiness	0.13 [0.00, 0.36]	0.45 [0.00, 1.05]	1.25 [0.68, 1.88]	− 0.80 [− 1.65, 0.10]
Sadness	0.84 [0.50, 1.17]	0.96 [0.38, 1.51]	1.42 [0.93, 1.90]	− 0.46 [− 1.18, 0.29]
Surprise	0.38 [0.09, 0.68]	0.22 [0.00, 0.55]	1.23 [0.81, 1.61]	− **1.01 [**− **1.51, **− **0.46]**

The last column indicates the same statistics regarding the difference between groups in this parameter.

*Note.* Credible differences in which the 95% HDI completely exclude the ROPE (0, 0) are boldfaced.

**Figure 3:**
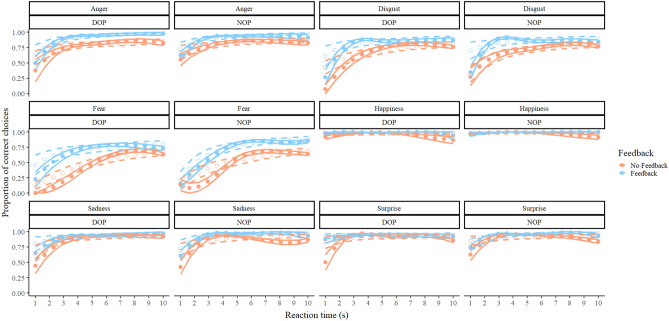
Real data (solid circles) and predictions of the model employed (blank circles) regarding the proportion of correct choices in each condition of the task as a function of emotion, group, feedback condition, and reaction time. For real data, values were aggregated in 15 bins of equal length ranging from 1 to 10 s. The predictions employed the number on the centre of each bin as input. Solid lines represent the standard error of the mean (for real data) and dashed lines represent the lower and upper limits of the 95% HDIs (for the predictions).

The *β*_*RT*_ parameter was credibly smaller for the DOP group than for the NOP group in two of the emotions, suggesting that the penalty for not seeing these emotions at full intensity was lower in the DOP condition than in the NOP condition. These emotions were Fear (mean of the posterior = − 0.91, 95% HDI from − 1.47 to − 0.34), and Surprise (mean of the posterior = − 1.01, 95% HDI from − 1.51 to − 0.46). Figure 3 shows the real data and the predictions of our model depending on the reaction time.

#### Learning curve (β_Trial_ parameter)

Concerning the estimation of learning during any condition, our interest is, on the one hand, to explore which conditions produced learning and, on the other hand, to check if learning achieved between conditions providing feedback is different for the same emotion. The *β*_*Trial*_ parameter directly informs about the difference in the probability of making a correct response (in log-odds) between the first and the last trial of each condition, so we will set as ROPE the minimum necessary value to modify this probability by 5%. The reason is similar to the one explained above. If there is an average change of less than half a correct response per individual when we compare the first and the last trial of each condition, learning will be considered negligible for that condition. This value is approximately 0.2 (when the comparison log-odds value is 0), so the ROPE for considering differences as credible for this parameter will be (− 0.2, 0.2). Results reporting the values for the parameter *β*_*Trial*_ are shown in Table 3.

**Table 3:** Means of the posteriors (and 95% HDIs) of the parameter *β*_*Trial*_ in each feedback condition.

	Feedback condition			Differences		
	No feedback	DOP	NOP	No feedback vs. feedback NOP	No feedback vs. feedback DOP	Feedback DOP vs. feedback NOP
Anger	− **0.75 [**− **1.09, **− **0.42]**	− 0.66 [− 1.31, 0.03]	− 0.43 [− 1.09, 0.21]	− 0.32 [− 1.09, 0.39]	− 0.09 [− 0.84, 0.64]	− 0.23 [− 1.18, 0.68]
Disgust	− **0.66 [**− **0.96, **− **0.34]**	− 0.58 [− 1.09, − 0.05]	− 0.44 [− 0.96, 0.10]	− 0.21 [− 0.83, 0.37]	− 0.07 [− 0.68, 0.53]	− 0.14 [− 0.87, 0.61]
Fear	− **1.18 [**− **1.47, **− **0.88]**	− **0.71 [**− **1.11, **− **0.25]**	− **0.81 [**− **1.29, **− **0.34]**	− 0.37 [− 0.92, 0.20]	− 0.47 [− 0.99, 0.06]	0.10 [− 0.56, 0.73]
Happiness	− 0.27 [− 1.10, 0.54]	0.97 [− 0.48, 2.47]	0.70 [− 0.73, 2.24]	− 0.97 [− 2.63, 0.75]	− 1.24 [− 2.98, 0.42]	0.27 [− 1.83, 2.33]
Sadness	− 0.61 [− 1.02, − 0.18]	− 0.18 [− 0.91, 0.56]	0.25 [− 0.69, 1.23]	− 0.86 [− 1.92, 0.16]	− 0.43 [− 1.28, 0.41]	− 0.43 [− 1.60, 0.79]
Surprise	− 0.33 [− 0.76, 0.13]	− 0.14 [− 0.91, 0.64]	0.28 [− 0.53, 1.08]	− 0.61 [− 1.51, 0.34]	− 0.18 [− 1.08, 0.70]	− 0.42 [− 1.55, 0.67]

The last three columns indicate the same statistics regarding the difference between feedback conditions in this parameter.

*Note.* Credible differences in which the 95% HDI completely exclude the ROPE (− 0.2, 0.2) are boldfaced.

**Figure 4:**
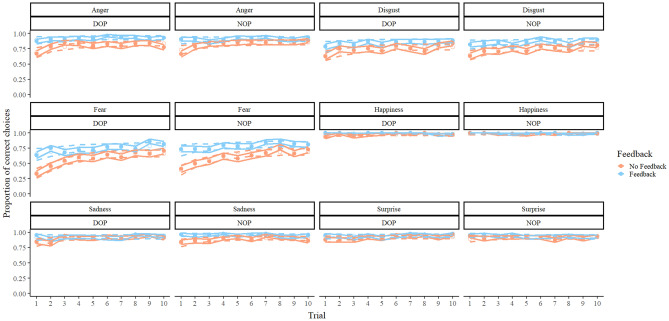
Real data (solid circles) and predictions of the model employed (blank circles) regarding the proportion of correct choices in each condition of the task as a function of emotion, group, feedback condition, and trial. Solid lines represent the standard error of the mean (for real data) and dashed lines represent the lower and upper limits of the 95% HDIs (for the predictions).

The *β*_*Trial*_ parameter suggested that when feedback was not received in earlier trials, the probability of making a correct response was lower for the emotions Anger (mean of the posterior = − 0.75, 95% HDI from − 1.09 to − 0.42), Disgust (mean of the posterior = − 0.66, 95% HDI from − 0.96 to − 0.34), and Fear (mean of the posterior = − 1.18, 95% HDI from − 1.47 to − 0.34). When feedback was received after the correct responses, the only emotion that suggested less probability of correct responses in earlier trials was Fear, for both the DOP group (mean of the posterior = − 0.71, 95% HDI from − 1.11 to − 0.25), and the NOP group (mean of the posterior = − 0.81, 95% HDI from − 1.29 to − 0.34). No credible differences were found between the different feedback conditions regarding the *β*_*Trial*_ parameter. Figure 4 shows the real data and the predictions of our model depending on the trial.

### Schizotypal personality traits

Descriptive statistics of the score in the SPQ of our participants, separately for each of the three factors and the total score, are presented in Table 4.

**Table 4:** Descriptive statistics of the score of each factor of the SPQ, as well as the total score, obtained by participants in our experiment.

Factor	M	SD	Min–Max	SK	K	Percentiles				
						10	25	50	75	90
Cognitive-Perceptual	9.53	5.90	0–24	0.38	-0.62	2.00	4.00	9.00	13.00	17.20
Interpersonal	11.94	6.34	0–27	0.26	− 0.77	4.00	7.00	12.00	16.00	20.20
Disorganized	4.80	3.63	0–14	0.60	− 0.51	0.80	2.00	5.00	7.00	10.00
Total score	23.53	11.37	0–53	0.14	− 0.50	9.00	15.00	23.00	32.00	36.20

*Note.* M, mean; SD, Standard Deviation; Min, Minimum; Max, Maximum; SK, Skewness; K, Kurtosis. The maximum possible score is 33 for the Cognitive-Perceptual factor, 33 for the Interpersonal factor, 16 for the Disorganized factor, and 74 for the Total score.

#### Effect of schizotypal personality traits levels on dynamic emotional facial expression recognition (β_CP_, β_IN_, β_DI_, β_CP_RT_, β_IN_RT_, β_DI_RT_, β_CP_Trial_, β_IN_Trial_, and β_DI_Trial_ parameters)

**Figure 5:**
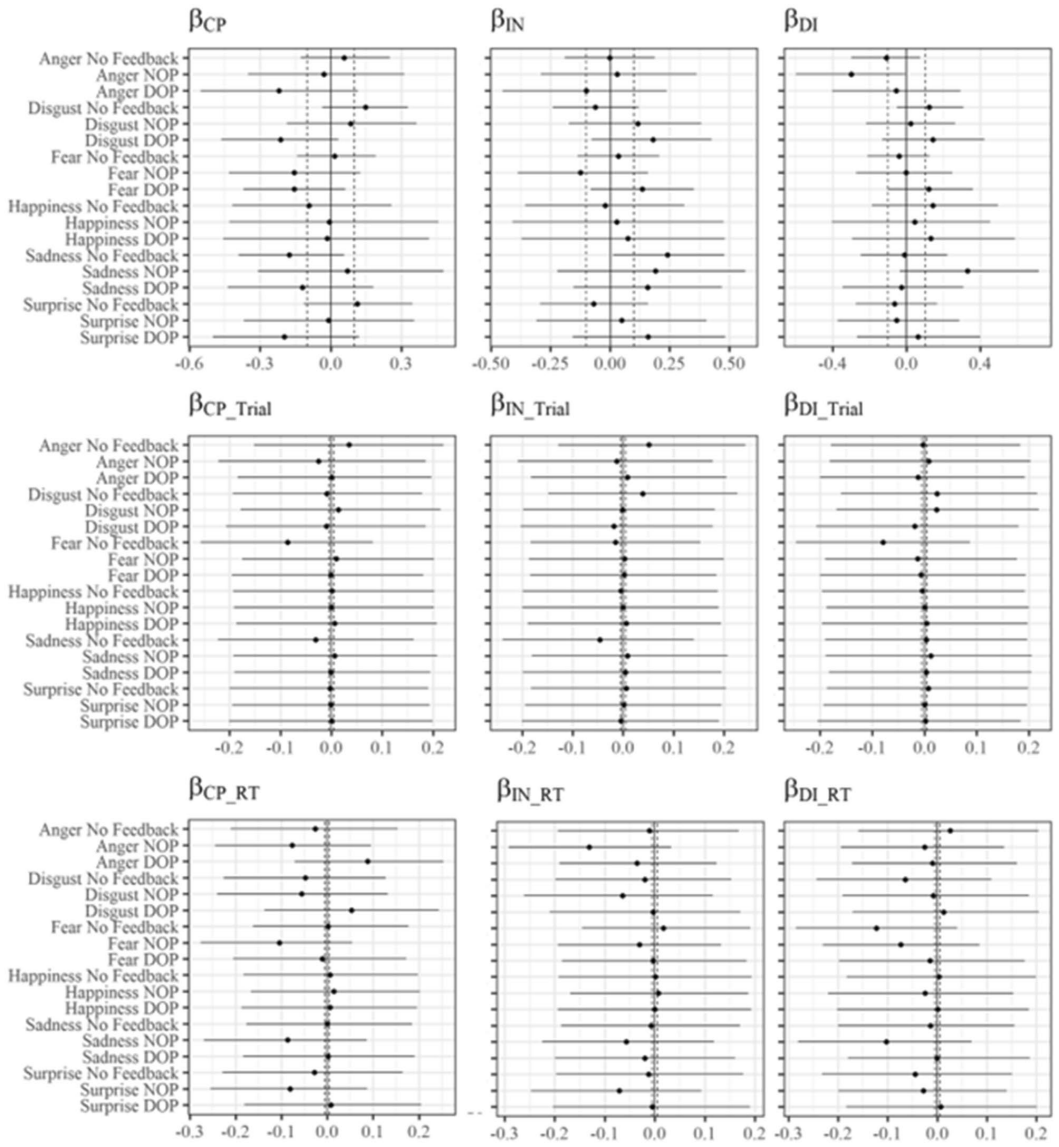
Values of the parameters involving the effect of schizotypal personality trait levels in our model. The points represent the mean of the posteriors of each estimation and the horizontal lines indicate the upper and lower limit of the 95% HDIs. The vertical solid line marks the 0 value, and the vertical dashed lines mark the interval employed as ROPE in each condition. *Note*. Parameters *β*_*CP*_,* β*_*IN*_, and* β*_*DI*_ account for the direct effect of the levels of each schizotypal personality factor in the probability of making a correct response. Parameters* β*_*CP_Trial*_,* β*_*IN_Trial*_, *β*_*DI_Trial*_, *β*_*CP_RT*_,* β*_*IN_RT*_, and *β*_*DI_RT*_ account for the moderative effect of the levels of each schizotypal personality factor in parameters *β*_*Trial*_ and *β*_*RT*_ respectively.

To estimate the direct effect of each schizotypal personality factor score on the probability of making a correct response in each condition *(β*_*CP*_,* β*_*IN*_, and* β*_*DI*_ parameters) we will set as ROPE the minimum necessary value to modify this probability by 5% when the standardized score of that schizotypal personality factor varies in 2 standard deviations (SDs). As explained above, the reason is that if there is an expected change of less than half a correct response in each condition when the standardized score in that schizotypal personality factor decreases or increases by 2 SDs, this change will be considered negligible. As this value is approximately 0.2 when the comparison log-odds value is 0, the ROPE for estimating these effects as negligible will be (− 0.1, 0.1), since parameter values would correspond to the change in log-odds when the predictor variable changes by one SD, as they are standardized. In contrast, to estimate the moderation effect that each schizotypal personality factor may exert on the parameters *β*_*Trial*_ and *β*_*RT*_ in each condition *(β*_*CP_Trial*_,* β*_*IN_Trial*_, *β*_*DI_Trial*_, *β*_*CP_RT*_,* β*_*IN_RT*_, and *β*_*DI_RT*_ parameters), the ROPE will correspond to the minimum necessary value to modify these effects by 1% when the standardized score of that schizotypal personality factor varies in 2 standard deviations (SDs), which would be the interval (− 0.005, 0.005). No direct or moderation effect of the levels of any schizotypal personality factor on any of the conditions was suggested by our data. Results are graphically presented in Fig. 5.

## Discussion

The present study explored the recognition of emotional facial expressions in virtually generated faces that morphed from a neutral expression to one of the six basic emotions, considering both the accuracy and the intensity of the facial expression of emotion when the response was produced. Our goals were to compare learning and performance in two groups using the NOP and the DOP to check whether the latter may grant greater learning or a more accurate recognition of emotions, and to explore if schizotypal personality trait levels may be negatively affecting the performance of our participants.

Regarding the first goal, we did not see any credible difference between the NOP and the DOP groups in the improvement in accuracy for presenting feedback compared to the trials where feedback was not presented. However, we have observed that when participants received feedback, those that underwent the DOP condition presented a lower reduction in their probability of making a correct response when the facial expressions of emotions fear and surprise were not shown at full intensity. This finding may suggest that the DOP is facilitating the recognition of these facial expressions of emotion when they are presented at subtler intensity levels, which may have important implications since, in daily life, emotions are suggested to be typically expressed in dynamic^37^ and subtle^61^ forms. Therefore, reducing the bias for not seeing an emotion at its highest intensity may have direct benefits on daily life for people that have problems recognising emotional facial expressions. Future studies could thus be carried out to explore possible improvements in clinical populations that feature impairments in facial emotion recognition.

As fear was the emotion that apparently was the hardest to identify, our previous prediction about the effect of the DOP being more notable in tasks that are more challenging is supported by the abovementioned results. It is also noteworthy that fear and surprise are usually confused with each other and their prototypical facial expressions share several AUs^62^. Following the FACS Investigator’s Guide^45^, most of the prototypical facial configurations of fear contain the same AUs as those of surprise, with the addition of AUs 4 and 20, which correspond to the brow lowerer and lip stretcher respectively. This study employed the FACS guidelines to generate stimuli, so this overlap between AUs also occurred. As the use of differential outcomes has classically been used to improve discriminative learning, perhaps it helped participants better discriminate between the AUs that differed between those two emotions.

Future studies may use eye-tracking approaches to explore if the implementation of the DOP is somehow facilitating this recognition of dynamic emotional facial expressions through a more efficient gazepath (e.g., focusing on the distinct facial regions that differ between them) or by extracting more useful information for regions they fixate on, which could be critical for the discrimination between fear and surprise. Furthermore, future studies may assess emotion recognition via different channels, as fear and surprise are mistaken not only regarding emotional facial expressions, but also when voices are used to discriminate emotions^63^. Exploring emotion recognition via other channels could be useful to determine whether this facilitation of the DOP is specific for facial expressions or if it is oriented to the discrimination of the emotions themselves. Moreover, the collection of additional physiological data could further enhance our understanding by assessing the level of vigilance during distinct conditions, allowing to explore whether vigilance levels influence accuracy.

It is crucial to mention that our results concerning most emotional facial expressions did not suggest any learning during feedback conditions. That is, performance when our participants received feedback after their correct responses was not credibly different when we compare their probability of making a correct response when a specific emotion is presented for the first time and for the last time during that block, suggesting there was no learning curve over these trials. The only exception was fear, which was also the one with the lowest percentage of correct responses. As we employ a non-clinical sample consisting of undergraduate students and their baseline performance for the recognition of almost all emotions could already be at a high level, with little room for improvement, these results could reflect ceiling effects. Employing different samples that could be impaired regarding this ability, such as patients diagnosed with schizophrenia^16,17^, may reveal different results, so studies using similar methodologies while expanding the target populations are encouraged.

Finally, it is also remarkable that when feedback was not received, a learning curve was found in three of the six emotions. This also manifests the importance of including enough practice trials when we are assessing our participants since, as they are not receiving feedback depending on their performance, practice effects could be the only expected explanation of their improvement over this condition. For this reason, comparing the average percentage of correct responses in blocks with and without feedback could lead to confounds in the interpretation of the results. Specifically, a more accurate estimation of the baseline performance of the participants would be their probability of making a correct response in the latter trial of the block without feedback. Employing an adequate approach regarding data analysis when training procedures are being compared is also encouraged for these reasons.

Concerning the second goal, we did not observe any credible effect of the levels of schizotypal personality traits in the recognition of dynamic emotional facial expressions. Additionally, we did not observe any moderation effect of these trait levels in either the influence that the intensity of the shown emotion, the trial, or the type of feedback provided may exert on the probability of making a correct response. As we mentioned in the introduction, even though some studies found an effect of schizotypal personality traits in the recognition of emotional facial expressions both in dynamic^27^ and static^28^ stimuli, other studies have not observed this effect^29–32^. Considering studies specifically employing dynamic stimuli, one investigation utilising video vignettes of real faces^27^ suggested a potential association between schizotypal personality traits and poorer recognition of emotional facial expressions. In contrast, another study employing morphed dynamic stimuli from virtual faces^29^ did not indicate such an effect. Our results align with the latter, suggesting that schizotypal personality trait levels may not be associated with the probability of correctly recognising dynamic emotional facial expressions. However, it is plausible that the type of stimuli may have influenced the observed outcomes. Future studies may further address this issue by comparing the effect of schizotypal personality traits in the recognition of emotional facial expressions when using both types of stimuli.

When literature is inconsistent regarding a specific topic, we consider it of great importance to take into account the size of the effects when making decisions and interpreting the results. The most usual way of making statistical decisions is comparing the value of the parameters of interest, such as a difference between means or a regression coefficient, exclusively with the value 0, and making dichotomic decisions about the existence (or absence) of effects based on this difference. Results regarding the study of psychiatric conditions and facial emotion identification typically present heterogeneity and it is usual that when an effect is found, the size is relatively small^64^. It could be possible that some of these effects arise from randomness or other methodological reasons, but more importantly, even if these small effects were genuine, it would be desirable to establish a minimum effect to-be-found that would be considered non-negligible. This may at least be slightly wider than zero depending on the conditions of the employed evaluation when it is possible. We believe that the use of tools for establishing this minimum criterion when making decisions, such as ROPEs^56^ employed in the present analyses, may help to prevent these inconsistencies, and they should be responsibly employed by researchers to avoid the biased publication of “positive” results, which especially affects the fields of psychiatry and psychology^65,66^.

Our study and its conclusions also present some limitations. The most important is that our most relevant finding, which is the suggested facilitating effect of the DOP to recognise fear and surprise when these are presented at less intensity, was not predicted. Future studies with similar methodologies must replicate this finding so inferences regarding this effect may be strengthened. Besides, our sample is limited to undergraduate students, so no inferences may be made regarding clinical populations and the effect that schizotypal personality trait levels may have on the recognition of emotional facial expressions when a clinical condition is also present. Additionally, there was a sex bias towards women in the composition of our sample, which is usually found in studies featuring mostly psychology students, such as the present research. Lastly, our study could benefit from a larger sample size, which would consequently result in narrower HDIs and could lead to more specific inferences about those data for which we withheld the decision, such as the possible effect of schizotypal personality traits in emotional facial expression recognition.

Further limitations inherent in our study are related to ecological validity. While our dynamic morphs may capture critical stages in the recognition of emotional facial expressions, the duration and frame rate of the videos used as stimuli may not accurately replicate the real-time dynamics of emotional facial expressions, which tend to unfold more rapidly^67^. Additionally, the linear movement introduced by the dynamic morph may lack authenticity in portraying the nuanced and non-linear nature of realistic facial expression changes. Besides, although virtual and real faces exhibit comparable recognition of expressed emotions in both healthy and clinical populations^39,40^, virtual faces have also been associated with challenges in memory retention^68^ and diminished effects of well-established phenomena, such as the other-race effect^69^, which could be attributed to the less realistic appearance of these stimuli.

Nevertheless, our results seem promising as they might suggest that a free-cost and easy-to-apply procedure could be employed to improve the probability of recognising some of the emotional facial expressions when they are expressed at subtler intensity levels in a sample of undergraduate students, though further research is required. As we expected that people presenting clinical conditions may show a poorer baseline performance regarding this ability^14,15^, we also predict that the beneficial effects of the DOP may be more notable in these populations, both in a greater facilitation of the recognition of emotions and in a larger number of emotions that benefit from this procedure. Future studies may test this hypothesis and hopefully improve their ability to recognise emotional facial expressions at subtler intensity levels, which may positively contribute to their independent living and social functioning capacity.

### Supplementary Information

Supplementary Table 1.

## Data Availability

The employed dataset is publicly available at the Open Science Framework (OSF) repository (https://osf.io/ygpx9/?view_only=dbdbe28eb8554451bf90e4ff69f357c4).
