# Supplementary material for: The role of the differential outcomes procedure and schizotypy in the recognition of dynamic facial expressions of emotions
Source: Sci Rep. 2024 Jan 28;14:2322. doi: 10.1038/s41598-024-52893-9 (PMC10822869; doi:10.1038/s41598-024-52893-9)
Supplement: Supplementary file 1 — Supplementary Table 1. [file 41598_2024_52893_MOESM1_ESM.docx]

**Supplementary table 1**

Mean ratings (and standard error of the mean) of representativeness of each combination of AUs for each of the basic emotions ranging from 0 (not representative at all) to 10 (totally representative). AUs chosen following the Facial Action Coding System (FACS) Investigator’s Guide (Ekman et al., 2002).

| Emotion  (based on FACS) | Representativeness rating | | | | | |
| --- | --- | --- | --- | --- | --- | --- |
| Anger |  |  |  |  |  |  |
| AUs | Anger | Disgust | Fear | Happiness | Sadness | Surprise |
| 4, 5 | 4.90 (0.14) | 0.47 (0.06) | 0.25 (0.05) | 0.06 (0.02) | 0.61 (0.08) | 0.11 (0.04) |
| 4, 5, 7, 10, 22, 23, 25 | 7.62 (0.12) | 1.73 (0.13) | 0.07 (0.03) | 0.01 (0.01) | 0.00 (0.00) | 0.24 (0.05) |
| **4, 5, 7, 10, 22, 23, 26** | **7.84 (0.13)** | 0.90 (0.10) | 0.24 (0.05) | 0.04 (0.03) | 0.08 (0.04) | 0.99 (0.11) |
| 4, 5, 7, 10, 23, 25 | 5.18 (0.17) | 2.92 (0.15) | 0.23 (0.05) | 0.00 (0.00) | 0.22 (0.05) | 0.42 (0.07) |
| 4, 5, 7, 10, 23, 26 | 6.46 (0.16) | 1.50 (0.13) | 0.54 (0.08) | 0.02 (0.02) | 0.14 (0.04) | 1.34 (0.12) |
| 4, 5, 7, 17, 23 | 6.42 (0.13) | 0.39 (0.06) | 0.16 (0.04) | 0.01 (0.01) | 1.14 (0.11) | 0.06 (0.03) |
| 4, 5, 7, 17, 24 | 6.44 (0.13) | 0.42 (0.06) | 0.08 (0.02) | 0.02 (0.02) | 0.65 (0.08) | 0.03 (0.01) |
| 4, 5, 7, 23 | 5.14 (0.14) | 0.37 (0.06) | 0.18 (0.04) | 0.18 (0.04) | 0.62 (0.08) | 0.09 (0.03) |
| 4, 5, 7, 24 | 5.06 (0.14) | 0.55 (0.07) | 0.11 (0.03) | 0.09 (0.03) | 0.49 (0.07) | 0.12 (0.04) |
| 4, 7 | 4.97 (0.14) | 0.81 (0.09) | 0.22 (0.05) | 0.08 (0.03) | 0.52 (0.07) | 0.12 (0.03) |
| 17, 24 | 0.63 (0.08) | 0.36 (0.06) | 0.17 (0.04) | 0.67 (0.08) | 1.85 (0.13) | 0.21 (0.05) |
|  |  |  |  |  |  |  |
| Disgust |  |  |  |  |  |  |
| AUs | Anger | Disgust | Fear | Happiness | Sadness | Surprise |
|  |  |  |  |  |  |  |
| 9 | 3.86 (0.15) | 1.96 (0.13) | 0.11 (0.04) | 0.21 (0.05) | 0.32 (0.06) | 0.07 (0.03) |
| 9, 15, 16 | 3.82 (0.16) | 2.97 (0.14) | 0.26 (0.05) | 0.05 (0.02) | 0.29 (0.05) | 0.36 (0.06) |
| 9, 16, 25 | 4.03 (0.16) | 2.40 (0.14) | 0.20 (0.05) | 0.20 (0.05) | 0.02 (0.01) | 0.57 (0.08) |
| 9, 16, 26 | 4.44 (0.17) | 0.93 (0.09) | 0.43 (0.07) | 0.04 (0.02) | 0.05 (0.02) | 2.73 (0.15) |
| 9, 17 | 5.43 (0.15) | 1.45 (0.11) | 0.06 (0.02) | 0.05 (0.02) | 0.41 (0.07) | 0.01 (0.00) |
| **10** | 0.81 (0.10) | **4.94 (0.15)** | 0.67 (0.09) | 0.18 (0.04) | 0.20 (0.04) | 0.59 (0.07) |
| 10, 16, 25 | 1.53 (0.13) | 3.07 (0.14) | 0.44 (0.07) | 0.67 (0.09) | 0.07 (0.03) | 0.99 (0.09) |
| 10, 16, 26 | 1.93 (0.14) | 1.29 (0.11) | 1.16 (0.11) | 0.16 (0.05) | 0.06 (0.02) | 3.96 (0.16) |
| 10, 17 | 3.24 (0.17) | 4.83 (0.16) | 0.27 (0.06) | 0.08 (0.03) | 0.08 (0.03) | 0.17 (0.04) |
|  |  |  |  |  |  |  |
| Fear |  |  |  |  |  |  |
| AUs | Anger | Disgust | Fear | Happiness | Sadness | Surprise |
| 1, 2, 4 | 0.78 (0.08) | 0.44 (0.06) | 0.53 (0.07) | 0.23 (0.05) | 2.88 (0.14) | 0.13 (0.03) |
| 1, 2, 4, 5 | 0.75 (0.08) | 0.31 (0.05) | 0.74 (0.08) | 0.24 (0.05) | 2.78 (0.14) | 0.19 (0.04) |
| 1, 2, 4, 5, 20 | 0.89 (0.09) | 0.38 (0.06) | 0.86 (0.09) | 0.03 (0.02) | 4.35 (0.15) | 0.10 (0.03) |
| 1, 2, 4, 5, 20, 25 | 0.65 (0.08) | 1.07 (0.10) | 1.82 (0.13) | 0.06 (0.03) | 3.04 (0.15) | 0.43 (0.06) |
| **1, 2, 4, 5, 20, 26** | 0.57 (0.08) | 1.66 (0.14) | **2.99 (0.16)** | 0.03 (0.02) | 1.18 (0.11) | 3.49 (0.16) |
| 1, 2, 4, 5, 20, 27 | 0.88 (0.10) | 0.98 (0.11) | 2.66 (0.16) | 0.01 (0.01) | 0.72 (0.09) | 4.31 (0.16) |
| 1, 2, 4, 5, 25 | 0.62 (0.08) | 0.56 (0.07) | 1.65 (0.12) | 0.17 (0.04) | 1.81 (0.12) | 1.53 (0.11) |
| 1, 2, 4, 5, 26 | 0.66 (0.08) | 0.50 (0.07) | 1.95 (0.14) | 0.04 (0.02) | 1.10 (0.10) | 5.19 (0.15) |
| 1, 2, 4, 5, 27 | 0.69 (0.09) | 0.41 (0.06) | 1.33 (0.12) | 0.17 (0.04) | 0.42 (0.07) | 6.08 (0.14) |
| 1, 2, 5 | 0.14 (0.04) | 0.16 (0.04) | 0.38 (0.06) | 0.88 (0.09) | 1.65 (0.12) | 1.25 (0.11) |
| 1, 2, 5, 25 | 0.09 (0.03) | 0.21 (0.04) | 0.84 (0.09) | 0.80 (0.09) | 0.96 (0.10) | 3.09 (0.15) |
| 1, 2, 5, 26 | 0.05 (0.02) | 0.15 (0.04) | 0.98 (0.10) | 0.18 (0.04) | 0.43 (0.07) | 6.57 (0.12) |
| 1, 2, 5, 27 | 0.03 (0.02) | 0.13 (0.04) | 0.47 (0.08) | 0.72 (0.09) | 0.18 (0.05) | 7.42 (0.11) |
| 5, 20 | 0.42 (0.07) | 0.49 (0.07) | 0.38 (0.06) | 0.55 (0.08) | 2.09 (0.13) | 0.30 (0.06) |
| 5, 20, 25 | 0.49 (0.07) | 1.05 (0.10) | 1.03 (0.10) | 0.52 (0.07) | 0.96 (0.09) | 0.97 (0.10) |
| 5, 20, 26 | 0.22 (0.05) | 1.32 (0.12) | 2.02 (0.14) | 0.13 (0.04) | 0.39 (0.07) | 4.36 (0.16) |
| 5, 20, 27 | 0.20 (0.05) | 0.50 (0.08) | 1.35 (0.12) | 0.26 (0.05) | 0.12 (0.04) | 5.58 (0.15) |
| 20 | 0.43 (0.06) | 0.58 (0.08) | 0.28 (0.06) | 0.58 (0.08) | 1.97 (0.12) | 0.19 (0.05) |
| Happiness |  |  |  |  |  |  |
| AUs | Anger | Disgust | Fear | Happiness | Sadness | Surprise |
| **6, 12** | 0.02 (0.01) | 0.06 (0.02) | 0.02 (0.01) | **8.00 (0.10)** | 0.13 (0.04) | 0.13 (0.04) |
| 12 | 0.02 (0.02) | 0.12 (0.03) | 0.02 (0.01) | 7.88 (0.11) | 0.09 (0.04) | 0.20 (0.05) |
|  |  |  |  |  |  |  |
| Sadness |  |  |  |  |  |  |
| AUs | Anger | Disgust | Fear | Happiness | Sadness | Surprise |
| 1 | 0.09 (0.03) | 0.13 (0.03) | 0.63 (0.08) | 0.43 (0.06) | 3.58 (0.15) | 0.30 (0.06) |
| 1, 4 | 0.45 (0.07) | 0.26 (0.05) | 0.77 (0.09) | 0.17 (0.04) | 4.96 (0.14) | 0.09 (0.03) |
| 1, 4, 11 | 0.35 (0.06) | 0.32 (0.05) | 0.85 (0.09) | 0.54 (0.07) | 4.31 (0.15) | 0.18 (0.04) |
| 1, 4, 15 | 0.27 (0.06) | 0.08 (0.03) | 0.49 (0.00) | 0.00 (0.00) | 7.13 (0.12) | 0.05 (0.02) |
| **1, 4, 15, 17** | 0.23 (0.05) | 0.12 (0.03) | 0.36 (0.06) | 0.09 (0.04) | **7.97 (0.11)** | 0.04 (0.02) |
| 6, 15 | 0.96 (0.10) | 0.76 (0.09) | 0.24 (0.05) | 0.29 (0.05) | 3.18 (0.15) | 0.16 (0.04) |
| 11, 17 | 0.46 (0.07) | 0.36 (0.06) | 0.18 (0.04) | 1.21 (0.11) | 0.93 (0.09) | 0.16 (0.04) |
|  |  |  |  |  |  |  |
| Surprise |  |  |  |  |  |  |
| AUs | Anger | Disgust | Fear | Happiness | Sadness | Surprise |
| 1, 2, 5 | 0.14 (0.04) | 0.16 (0.04) | 0.38 (0.06) | 0.88 (0.09) | 1.65 (0.12) | 1.25 (0.11) |
| 1, 2, 5, 26 | 0.05 (0.02) | 0.15 (0.04) | 0.98 (0.10) | 0.18 (0.04) | 0.43 (0.07) | 6.57 (0.12) |
| **1, 2, 5, 27** | 0.03 (0.02) | 0.13 (0.04) | 0.47 (0.08) | 0.72 (0.09) | 0.18 (0.05) | **7.42 (0.11)** |
| 1, 2, 26 | 0.06 (0.03) | 0.17 (0.04) | 1.15 (0.11) | 0.23 (0.05) | 0.33 (0.06) | 6.54 (0.13) |
| 1, 2, 27 | 0.00 (0.00) | 0.21 (0.05) | 0.49 (0.08) | 0.72 (0.08) | 0.15 (0.03) | 7.34 (0.12) |
| 5, 26 | 0.10 (0.03) | 0.21 (0.05) | 0.78 (0.09) | 0.22 (0.05) | 0.17 (0.04) | 6.12 (0.13) |
| 5, 27 | 0.07 (0.03) | 0.11 (0.03) | 0.35 (0.06) | 0.79 (0.09) | 0.07 (0.03) | 7.04 (0.12) |

*Note.* The combination of AUs that showed the highest mean rating for one specific emotion, as well as the rating for this emotion, is boldfaced.
